# Sentinel-based Surveillance of Coyotes to Detect Bovine Tuberculosis, Michigan

**DOI:** 10.3201/eid1412.071181

**Published:** 2008-12

**Authors:** Kurt C. VerCauteren, Todd C. Atwood, Thomas J. DeLiberto, Holly J. Smith, Justin S. Stevenson, Bruce V. Thomsen, Thomas Gidlewski, Janet Payeur

**Affiliations:** US Department of Agriculture–Animal and Plant Health Inspection Service (USDA–APHIS) Wildlife Services, Fort Collins, Colorado, USA (K.C. VerCauteren, T.C. Atwood, T.J. DeLiberto, H.J. Smith, J.S. Stevenson); USDA-APHIS Veterinary Services Laboratories, Ames, Iowa, USA (B.V. Thomsen, T. Gidlewski, J. Payeur)

**Keywords:** Bovine tuberculosis, *Canis latrans*, coyote, deer, *Mycobacterium bovis*, sentinel, surveillance, research

## Abstract

Bovine tuberculosis (TB) is endemic in white-tailed deer (*Odocoileus virginianus*) in the northeastern portion of Michigan’s Lower Peninsula. Bovine TB in deer and cattle has created immense financial consequences for the livestock industry and hunting public. Surveillance identified coyotes (*Canis latrans*) as potential bio-accumulators of *Mycobacterium bovis*, a finding that generated interest in their potential to serve as sentinels for monitoring disease risk. We sampled 175 coyotes in the bovine TB–endemic area. Fifty-eight tested positive, and infection prevalence by county ranged from 19% to 52% (statistical mean 33%, SE 0.07). By contrast, prevalence in deer (n = 3,817) was lower (i.e., 1.49%; Mann-Whitney U_4,4_ = 14, p<0.001). By focusing on coyotes rather than deer, we sampled 97% fewer individuals and increased the likelihood of detecting *M. bovis* by 40%. As a result of reduced sampling intensity, sentinel coyote surveys have the potential to be practical indicators of *M. bovis* presence in wildlife and livestock.

## Abstract 2

Coyotes could be used as sentinels to detect *Mycobacterium bovis* in the wild.

The emergence and reemergence of zoonotic diseases are becoming increasingly important issues for numerous reasons, including deforestation and habitat fragmentation, increased globalization of travel and trade, urbanization, and bioterrorism concerns. Diseases such as severe acute respiratory syndrome (SARS), avian influenza, transmissible spongiform encephalopathies, Rift Valley fever, West Nile disease, anthrax, and *Escherichia coli* O157 infections recently have resulted in major public health and economic concerns, as well as public anxiety. Over 60% of the 1,415 known human pathogens and 75% of the 175 emerging pathogens are zoonotic (*1*). Many emerging diseases have spilled over from wildlife directly (e.g., West Nile virus infection, hantavirus infection, and Lyme disease) or indirectly through domestic or peridomestic species (e.g., avian influenza, SARS, and Nipah virus infections, plague) (*2*). Early detection of new disease outbreaks in domestic and wild animals is an essential prerequisite of disease control and eradication. Development of methods for early detection of diseases in free-ranging wildlife is problematic.

Development of practical strategies for conducting surveillance in free-ranging wildlife to detect and monitor disease and evaluate control efforts is a necessary component of predicting and managing emerging zoonoses. A case in point is bovine tuberculosis (TB). *Mycobacterium bovis,* the bacterial pathogen that causes bovine TB, has been identified in wildlife, domestic animals, and humans (*3*–*6*). Transmission of *M. bovis* may occur through ingestion of infected tissues or, less likely, through inhalation of aerosolized bacilli (*7*); typically, granulomatous lesions develop in the thoracic lymph nodes and lung after aerosol exposure, and granulomatous lesions develop in the abdominal lymph nodes after oral exposure. Bovine TB often progresses slowly, and clinical symptoms may not appear until advanced stages are reached (*8*,*9*). In 1995, *M. bovis* was found in free-ranging white-tailed deer (*Odocoileus virginianus*) in a localized area in the northeastern Lower Peninsula of Michigan (*10*). In subsequent years, a reemergence of *M. bovis* in Michigan cattle was detected; deer were postulated to be the source of infection. Because the socioeconomic impact of this discovery has been immense (*11*), a strategy was developed and implemented to monitor and eradicate *M. bovis* from wildlife and cattle. Although the strategy successfully reduced the apparent prevalence of *M. bovis* in deer, the disease still persists at low levels (e.g., 2001–2006 statistical mean 2.3%) because of high deer densities (statistical mean 13/km^2^) and spatiotemporal crowding resulting from supplemental feeding (*12*). As prevalence of *M. bovis* in deer decreases, the sample size required to detect positive deer increases, making monitoring of the disease in deer more difficult and costly. Eventually, prevalence in deer may become too low to accurately estimate through current methods because of the difficulty and expense of obtaining a sufficient sample size, and consequent difficulty of verifying disease eradication. We hypothesized that the presence of *M. bovis* in wild deer at low prevalence could be more accurately determined through an indirect estimator (i.e., a sentinel species).

Use of sentinel animals has been suggested as a cost-effective way to infer prevalence in host populations when direct estimation in such populations is difficult (*13*). As facultative scavengers, coyotes (*Canis latrans*) may act as biological sensors and bio-accumulators of *M. bovis*. by consuming infected host material, resulting in high rates of infection. Furthermore, social foraging by coyote populations (*14*,*15*) should increase the likelihood of multiple coyotes ingesting infected tissue from the same *M. bovis–*positive deer. As a logical corollary, the increased numeric exposure of coyotes to *M. bovis* should mediate an increased detection probability relative to sampling effort. Support for this hypothesis was provided by research (*5*), which reported an apparent prevalence of *M. bovis*. in opportunistically sampled coyotes as 4% in the general area where apparent prevalence in deer averaged 2.3% from 1995 through 2001 (*16*). Finally, coyote home-range sizes (statistical mean 14.25 km^2^, 95% confidence interval [CI] 9.54–18.96 km^2^) in Michigan allow for reasonable estimates of where infection was acquired (*17*).

We report on a sentinel-based surveillance program designed to detect *M. bovis* in coyotes. Specifically, we sought to determine whether 1) *M. bovis* occurrence in coyotes was detectable, given reduced sampling intensity relative to white-tailed deer, and 2) prevalence of *M. bovis* was greater in coyotes than deer for a given area. If so, coyotes should be effectual sentinels of *M. bovis* occurrence in free-ranging white-tailed deer.

## Methods

**Figure 1:**
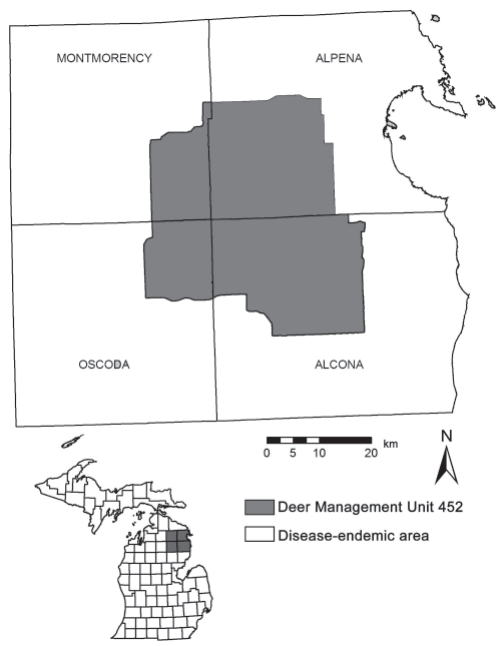
Coyote study area in Montmorency, Alpena, Alcona, and Oscoda Counties in the northeastern Lower Peninsula of Michigan, United States.

We worked within the 4-county bovine TB–endemic area in Michigan’s Lower Peninsula, where cattle herds continue to be infected and intensive sampling of hunter-killed deer is ongoing (Deer Management Unit [DMU] 452; Figure 1). DMU 452 is the historic core bovine TB–endemic area and remains a focal site of intensive sampling of hunter-killed deer (*18*). Prior carnivore surveillance conducted by the Michigan Department of Natural Resources (MDNR) had detected *M. bovis* in 18 of 249 sampled coyotes. Given the history of intensive surveillance and elevated *M. bovis* prevalence in the area, it was the logical choice to implement and evaluate a sentinel-based surveillance program. Habitat associations within DMU 452 were diverse; moraine uplands were dominated by forests of jack pine (*Pinus banksiana*), white pine (*P. alba*), oak (*Quercus* spp.), and maple (*Acer* spp*.*). Dominant lowland vegetation included tag alder (*Alnus rugosa*) and white cedar (*Thuja occidentalis*), and wetland ephemera were common. Annual precipitation typically ranged from 71 cm to 91 cm; most occurred as snowfall (*19*). Mean yearly summer and winter temperatures were 21°C and –10°C, respectively (*19*).

We trapped coyotes from December 2003 through September 2005 using padded foot-hold traps and scent lures in 15 townships within the 4-county area. We trapped coyotes in 6 townships in Alcona County, 5 in Oscoda County, 2 in Montmorency County, and 2 in Alpena County. Because a large proportion of land in the study area was privately owned (e.g., commercial hunting clubs, agricultural operations, residential development), landowner permission to access property dictated trap placement. Thus, we were unable to randomize trapping locations or distribute traps proportionally among counties. Within each township, traps were checked daily, and trapping was terminated when 10 coyotes were collected. Because multiple captures could occur on the final day of trapping, we occasionally collected >10 coyotes/township. We killed trapped coyotes with a 0.22-caliber gunshot to the brain, determined their age on the basis of tooth wear and eruption (*20*), and performed necropsy examinations on them within an hour of death to minimize autolysis. Tissues containing visible lesions as well as the parotid, mandibular, retropharyngeal, bronchial, mediastinal, and mesenteric lymph nodes were collected and submitted in formalin for histologic examination and fresh for mycobacterial culture.

Coyote samples were processed by following protocols used for histologic examination and mycobacterial culture of white-tailed deer samples (*6*,*10*). Fresh tissues for bacterial culture were digested and decontaminated with a sodium-hypochlorite-sodium hydroxide method (*21*). We then spun tissue suspensions in a refrigerated centrifuge at 6,000 × *g* for 20 minutes (*21*). Half of the supernatant was discarded, and the pellet was resuspended and swabbed on the following solid media: Middlebrook 7H10 agar containing sodium pyruvate (National Veterinary Services Laboratory [NVSL], Ames, IA, USA), Middlebrook 7H11 agar containing sodium pyruvate (NVSL), BBL Mycobactosel L-J medium slant (Becton Dickinson, Sparks, NJ, USA) and Middlebrook 7H11 with aspartic acid and pyruvate (Becton Dickinson) (*22*). We then injected the suspension (0.5 mL) into BACTEC 12 B liquid culture vials (Becton Dickinson) and BACTEC MGIT liquid culture tubes (Becton Dickinson) (*21*). The solid media tubes were incubated at 37 ± 2°C in a 10% CO_2_ incubator and examined weekly until colonies were observed or until an incubation period of 8 weeks was complete, at which time tubes with no growth were discarded (*21*). We incubated the BACTEC 12 B vials at 37 ± 2°C and monitored them in the BACTEC 460 instrument for 6 weeks (*21*,*22*). We incubated MGIT 960 tubes at 37 ± 2°C and monitored them in the BACTEC MGIT 960 instrument for 6 weeks (*21*–*23*). Colonies from solid media and liquid culture bottles that showed positive signals were confirmed as *M. tuberculosis* complex identification by a combination of Ziehl-Neelsen acid-fast staining and the AccuProbe *M. tuberculosis* complex nucleic acid probes (Gen-Probe, San Diego, CA, USA) (*21*,*23*). We then used niacin and nitrate biochemical tests to distinguish *M. bovis* from *M. tuberculosis* isolates (*21*,*23*).

Formalin-fixed tissues were processed and stained with hematoxylin and eosin. Any granulomatous lesions were then stained with a modified Ziehl-Neelson procedure and an auramine orange and acridine orange procedure (*24*,*25*). When tissues were identified as having granulomatous lesions and acid-fast bacilli, they were further evaluated by PCR. The PCR was performed on the formalin-fixed, paraffin-embedded tissue by using primers for IS*6110* to identify *M. tuberculosis* complex species, which include *M. bovis*, and 16S rRNA to identify *M. avium* complex species. The PCR procedures were similar to those described previously (*22*). Animals were considered positive if bacterial cultures isolated *M. bovis* from fresh tissues and/or fixed tissues had granulomatous lesions with acid-fast bacilli that were PCR positive for IS*6110*. All histologic screenings and PCRs were conducted at NVSL.

We used a log-linear model (*26*) to determine whether the count of *M. bovis–*positive coyotes was independent among age classes and sexes. We used adjusted residuals for describing and making inferences about the true association structure among the response variables. We used a Mann-Whitney test (*27*) to compare prevalence of *M. bovis* in coyotes to white-tailed deer sampled by MDNR during the same period.

## Results

We captured and collected tissues from 175 coyotes (91 males, 84 females) in 15 townships (statistical mean 11 coyotes/township, SE = 0.63) within DMU 452 and 14 control coyotes (8 males, 6 females) from Michigan’s Upper Peninsula. For coyotes sampled from DMU 452, we were able to classify 101 (51 males, 50 females) as juveniles (<2 years old) and 67 (34 males, 33 females) as adults. Age data were not collected from control coyotes. We identified 58 *M. bovis–*positive coyotes from DMU 452; 16 (28%) positive coyotes were trapped within the boundaries of property owned by 7 private hunt clubs distributed throughout DMU 452. Seven coyotes (5 males, 2 females) whose age could not be determined were negative for *M. bovis*. All control coyotes were negative for *M. bovis*, and they were not included in subsequent analyses or summary statistics. Unweaned pups were not sampled.

**Figure 2:**
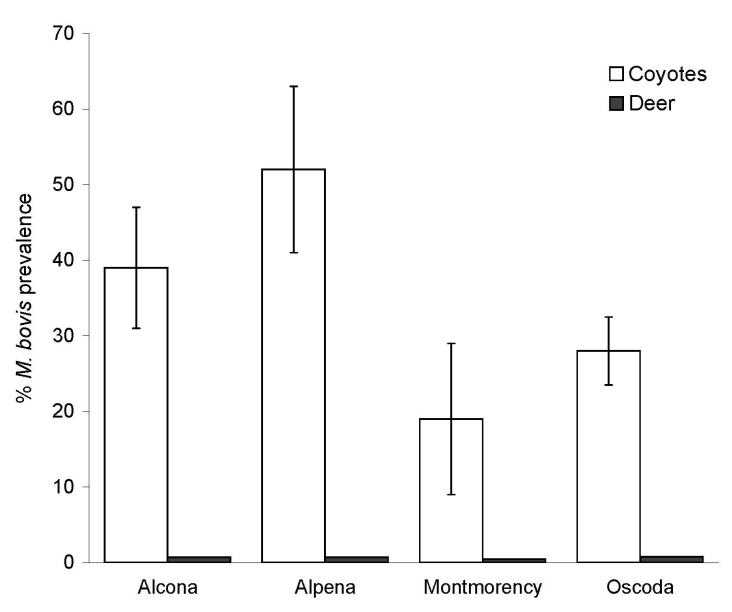
Percent prevalence of *Mycobacterium bovis–*positive coyotes (*Canis latrans*) and white-tailed deer (*Odocoileus virginianus*) in Montmorency, Alpena, Alcona, and Oscoda Counties, Michigan, 2003–2005. Prevalence estimates for white-tailed deer are expressed as a mean calculated from discrete sampling periods conducted in 2003, 2004, and 2005. Error bars for coyote estimates represent the standard error of the mean calculated across townships for each county. Estimates of *M. bovis* prevalence for white-tailed deer were not available for individual townships; standard errors were not calculated for counties.

Apparent prevalence of *M. bovis* infection did not differ by age (χ^2^ = 3.16, degrees of freedom [df] = 1, p = 0.07) or sex (χ^2^ = 0.05, df = 1, p = 0.83) class (log linear model; *26*). Percent prevalence of *M. bovis* was highest for coyotes sampled from Alpena County, followed by Alcona, Oscoda, and Montmorency Counties, respectively (Figure 2). Mean prevalence for the 4-county area was estimated at 33% (SE = 0.07; bovine TB–positive coyotes: n_Alcona_ = 23, n_Oscoda_ = 18, n_Alpena_ = 10, n_Montmorency_ = 7; Table). During the same period, MDNR identified 57 (1.49%) *M. bovis–*positive deer from a sample of 3,817 killed by hunters within DMU 452, and apparent prevalence was highest in Oscoda County, followed by Alcona, Alpena, and Montmorency Counties (Figure 2) (*18*). Mean apparent prevalence was significantly greater in coyotes than in deer (Mann-Whitney U_4,4_ = 14, p<0.001); this overall trend was consistent for all 4 counties. The proportion of *M. bovis–*positive deer sampled from DMU 452 during 2004–2005 fell within 95% confidence limits generated by calculating the proportion of positive deer from 1996 through 2003.

**Table:** Number of coyotes sampled and determined to be *Mycobacterium bovis* positive,* 4 counties, Michigan, USA, 2003–2005

County	No. sampled (no. positive)			
	Adult M	Adult F	Juvenile M	Juvenile F
Montmorency	6 (1)	8 (2)	6 (3)	5 (1)
Alpena	5 (2)	4 (2)	7 (5)	6 (1)
Alcona	11 (5)	12 (5)	16 (5)	20 (8)
Oscoda	12 (7)	9 (4)	23 (6)	18 (1)

*Determined by histologic examination and mycobacterial culture, followed by PCR for strain identification.

*M. bovis* (n = 58) was the most common mycobacterium isolated, but *M. avium* complex species (n = 12), *M. intracellulare* (n = 1), and *M. kansasii* (n = 1) were also identified by culture. *M. bovis* was the most common mycobacterial isolate found within the mesenteric lymph nodes. In 31 positive cases in which anatomic location of lymph nodes was identified, 14 animals were positive only in mesenteric lymph nodes, 14 were positive in both mesenteric and combined head and thoracic lymph nodes, and 3 animals were positive only in combined head and thoracic lymph nodes. No coyotes were detected concurrently infected with multiple *Mycobacterium* types.

**Figure 3:**
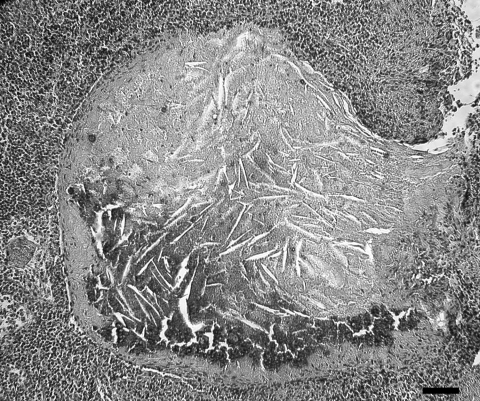
Granulomatous lymphadenitis caused by *Mycobacterium bovis* in a coyote (*Canis latrans*). The granulomas consist of a large central necrotic area with mineralization and cholesterol clefts surrounded by a thin rim of primarily macrophages and fibrous connective tissue. Scale bar = 55 μm.

**Figure 4:**
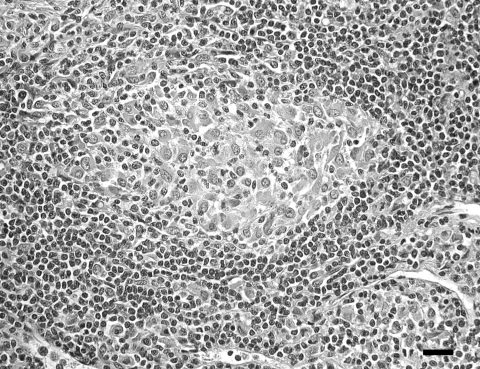
Focal histiocytic lymphadenitis caused by *Mycobacterium bovis* in a coyote (*Canis latrans*). Note the small, poorly delineated, aggregates of primarily macrophages within the lymph node cortex. Scale bar = 25 μm.

Lymph node lesions caused by *M. bovis* varied from focal to multifocal and ranged in size from 1 to 15 mm. Frequently, an affected lymph node contained several 1- to 5-mm granulomas. A single animal was found with multiple, large, 1- to 1.5-cm granulomas within the liver, lungs, pleura, and mesenteric lymph nodes. Microscopically, both lesions and the number of acid-fast bacilli within lesions were variable. Most lesions contained occasional acid-fast bacilli with fewer lesions containing numerous acid-fast bacilli. The most common microscopic lesion was a granuloma in the cortex of lymph nodes with large central areas of acellular, eosinophilic debris, with or without basophilic mineralized debris, and numerous cholesterol clefts. Necrotic debris was surrounded by a thin rim of macrophages, epithelioid macrophages, fibrous connective tissue, lymphocytes, only a few neutrophils, and plasma cells. Multinucleated giant cells were infrequent or absent (Figure 3). Less commonly, in some granulomas the central area of necrotic debris was almost entirely mineralized. A second type of lesion found in the cortex of the lymph nodes consisted only of small, poorly delineated aggregates of macrophages and epithelioid macrophages intermixed with low numbers of lymphocytes. In some animals, these small aggregates of macrophages were the only lesions identified (Figure 4).

## Discussion

We demonstrated the potential of using coyotes as sentinels to detect *M. bovis* occurrence in an area containing endemically infected white-tailed deer with a prevalence of <2%. By focusing on coyotes rather than deer, we sampled 97% fewer animals and detected a similar number of *M. bovis–*positive animals (i.e., 58 *M. bovis–*positive coyotes; 57 *M. bovis–*positive deer), which increased detection of *M. bovis* by 40%. Smaller samples mean less expense associated with laboratory testing. Moreover, smaller samples can result in shorter times between end of sampling and disease confirmation and therefore can increase opportunities for rapid disease management response.

Early in the study, we discovered the importance of collecting diagnostic samples as soon as possible after death. Rapid autolysis of the gastrointestinal tract and associated mesenteric nodes quickly minimizes the utility of these tissues for histologic and microbiologic examination. Delays between time of euthanasia and tissue collection reduced the ability to identify lesions and associated acid-fast organisms as well as to propagate the organism in culture and consequently lower the apparent incidence of disease. Related to this, because MDNR only submitted diagnostic samples from deer with visible lesions and because samples collected from deer were not taken as quickly after death as those from coyotes, the prevalence rates in deer may have been underestimated.

Also, infection of coyotes was independent of age groups and sex, which suggested that our sampling design did not bias detection of *M. bovis* occurrence relative to coyote demographic characteristics. This finding is critically important as to whether focal species are considered effectual disease sentinels (*28*) because age- or sex-biased dispersal can severely confound attempts to correlate the spatial distribution of disease occurrence between the sentinel and host. Capture biases in wildlife studies can be a legitimate concern, particularly where complex social behavior, such as agonism, can differentially influence the vulnerability of animals to various methods of capture. Our decision to collect coyotes exclusively by means of foot-hold traps, rather than hunting with dogs or with predator calls (the methods preferred by sport hunters), should have minimized sampling bias: socially dominant individual animals are potentially more susceptible to predator calls (*29*). Furthermore, standardizing sampling effort to a single trapping period with a goal of 10 animals/transect should have ensured that the animals that were captured, and their disease status, were representative of the at-large population (*30*).

Additional bias could accrue if infirmity influenced the probability of capture, thereby resulting in over- or underestimates of apparent prevalence (*31*–*33*). However, TB is a chronic infection, and animals usually survive in relatively good condition until severe clinical symptoms, such as extreme malaise (*8*), appear at the penultimate stage of disease (*9*). Because of this, there is a relatively short temporal frame (≈2 weeks) between the onset of moribund condition and death (*9*) when capture probabilities may be biased by disease status. We found no evidence of physical debilitation positively or negatively influencing capture probability. Of 58 *M. bovis–*positive coyotes captured, none showed symptoms of severe emaciation or lethargy suggestive of advanced disease, and only 1 coyote bore widely disseminated lesions visible on gross inspection during necropsy. Thus, we believe our trap-transect method of sampling coyotes was robust to potential bias associated with coyote disease status. Because the animals were euthanized upon capture, our work was not replicable. Therefore, we could not use a design based on mark-recapture to determine if, in fact, our sampling protocol produced stable, increasing, or diminishing prevalence estimates over successive trapping sessions.

It appears that for coyotes infected with *M. bovis*, lesions predominantly localized to the lymphoid tissue of the gastrointestinal tract, although lesions concurrently developed in lymph nodes of the head in 16 coyotes. Lesions ranged from acute to chronic; marked fibrosis and few acid-fast organisms were noted in the chronic lesions. Only 1 animal had evidence of advanced disease, as evidenced by lesions in the lung and liver, which may have been caused by a large infectious dose, a compromised immune system, or long-term infection. The spectrum and locations of lesions led us to postulate that coyotes may acquire *M. bovis* orally and have the immunologic ability to minimize and possibly eliminate the bacteria. Our study was not designed to determine route of transmission or whether coyotes were a maintenance reservoir for *M. bovis*. However, preliminary results of current research indicate that excretion of *M. bovis* by coyotes experimentally inoculated with oral doses (ranging from 10 to 10^5^ CFU) is probably unlikely or undetectable (M. Dunbar, National Wildlife Research Center, pers. comm.). If excretion of *M. bovis* is not likely in orally inoculated coyotes, then it is not likely to result in widespread infection among coyotes that would have become infected by ingesting infected tissue. Moreover, the absence of *M. bovis* in control coyotes sampled from the Upper Peninsula, where bovine TB has not been detected in white-tailed deer or cattle (*12*), lends further credence to the belief that coyotes are spillover rather than maintenance hosts.

For agrarian areas where livestock operations predominate, regular testing of domestic animals and slaughter of reactors can effectively prevent the long-term maintenance of *M. bovis* within localized livestock (*34*). However, in areas where livestock densities are low, *M. bovis* prevalence in wildlife must be surveyed directly (*13*). The disparity in prevalence relative to sampling effort between coyotes and deer is strong evidence that coyotes could be useful for monitoring *M. bovis* occurrence in Michigan (*4*). Coyotes in Michigan generally have larger home ranges than deer (coyotes, statistical mean 14.25 km^2^; white-tailed deer, statistical mean 2.11 km^2^; *17**,**35*) and appear to have a much higher per capita probability of developing detectable infection. However, because of discrepant home-range sizes, attempts to spatially correlate sources of infection for coyotes, sympatric wildlife, and domestic livestock will be confounded by spatial scale. Thus, some question about the source of infection in coyotes will always remain; the presence of an infected coyote can only provide a broad indication of the location of the original source of infection. Although we noted that 44% of all *M. bovis–*positive coyotes were trapped within the boundaries of private hunt clubs, we cannot infer that coyotes acquired the pathogen within club boundaries. The only way to circumvent this inferential deficit is to gather spatial information on a large sample of animals before killing them to determine their infection status (*13*) and then to develop probabilistic resource selection models (*36*).

As with other tools (e.g., radio transmitters, global positioning systems) and techniques (e.g., telemetry, population estimation), the sentinel species concept may not be applicable in some instances. For example, others have followed our lead to investigate the feasibility of using coyotes as sentinels for *M. bovis* in Manitoba, Canada, without documenting *M. bovis* in coyotes (*37*). Their results could have occurred because prevalence rates in cervids were so low that they were not detected, given the number of coyotes sampled; coyotes are not the appropriate sentinel species; or both. Just as it is useful to determine why coyotes can function well as sentinels in Michigan, it is valuable to point out why the same does not appear so in Manitoba. We concur with the authors of the Manitoba study (*37*) that their negative results could be due to 1) the fact that it was unknown if trapped coyote ranges overlapped cervid ranges (much less if they overlapped the ranges of potentially infected cervids), 2) too low coyote sample size relative to prevalence rate in cervids, and 3) coyotes not being likely to prey on elk (*Cervus elaphus*); if they scavenge kills of other predators (wolves [*Canis lupus*], black bears [*Ursus americanus*]; which may be appropriate sentinels in Manitoba), infected tissues are likely no longer present (*38*). Other reasons for their negative results could include the following: 1) ranges and diets of coyotes in the area were unknown, 2) the prevalence rate for cervids during the life of most coyotes collected was unknown and likely very low (<0.1%), and 3) if sample quality from carcasses salvaged from trappers or collected opportunistically was compromised, it could negatively affect the ability to detect *M. bovis*.

The potential benefits of using coyotes as sentinels for *M. bovis* occurrence ultimately relate to increased sampling efficiency and disease detection. Our work shows that coyotes are sensitive indicators of disease presence in Michigan. The collection protocol we designed to sample coyotes ensured the likelihood that sampled individuals were representative of the population and estimates of disease prevalence were relatively bias-free. Sentinel coyote surveys appear to be effectual cost- and labor-sensitive indicators of *M. bovis* presence in sympatric wildlife and domestic livestock. We concur with others (*1*,*28*) who endorse the use of sentinel-based surveillance programs, particularly when project goals include monitoring spatiotemporal changes in disease risk. In addition, we believe sentinel-based programs could facilitate adaptive monitoring of disease occurrence where the likelihood of horizontal transmission is great and/or spatial epidemiology is uncertain. From another perspective (*39*), we also believe that wildlife can serve as effective biologic sensors and satellites of some infectious disease epidemics and bioterrorism that threaten human health and safety.
